# Plasma concentrations of eleven cannabinoids in cattle following oral administration of industrial hemp (*Cannabis sativa*)

**DOI:** 10.1038/s41598-020-69768-4

**Published:** 2020-07-29

**Authors:** Michael D. Kleinhenz, Geraldine Magnin, Zhoumeng Lin, Jason Griffin, Katie E. Kleinhenz, Shawnee Montgomery, Andrew Curtis, Miriam Martin, Johann F. Coetzee

**Affiliations:** 1 Department of Clinical Sciences, College of Veterinary Medicine, Kansas State University, 1800 Denison Ave., Manhattan, KS 66502 USA; 2 Department of Anatomy and Physiology, College of Veterinary Medicine, Kansas State University, 1800 Denison Ave., Manhattan, KS 66502 USA; 3 Institute of Computational Comparative Medicine (ICCM), Kansas State University, 1800 Denison Ave., Manhattan, KS 66502 USA; 4 John C. Pair Horticulture Center, Kansas State University, 1901 East 95th St South, Haysville, KS 67060 USA; 5 Veterinary Diagnostic Laboratory, College of Veterinary Medicine, Kansas State University, 1800 Denison Ave., Manhattan, KS 66502 USA

**Keywords:** Therapeutics, Translational research

## Abstract

Cannabinoid production for medicinal purposes has renewed interest in utilizing byproducts of industrial hemp (IH) as a feed source for livestock. However, the presence of bioactive residues in animal tissues may pose a risk to consumers. The purpose of this study was to characterize the plasma pharmacokinetics (PK) of cannabinoids and their metabolites in cattle after a single oral exposure to IH. Eight castrated male Holstein calves received a single oral dose of 35 g of IH to achieve a target dose of 5.4 mg/kg cannabidiolic acid (CBDA). Blood samples were collected for 96 h after dosing. Plasma cannabinoid concentrations were profiled using liquid chromatography coupled with mass-spectroscopy (UPLC) and PK parameters were calculated using noncompartmental methods. The cannabinoids CBDA, tetrahydrocannabinolic acid-A (THCA-A), cannabidivarinic acid (CBDVA), and cannabichromenic acid (CBCA) were detected in all cattle after IH dosing. The geometric mean maximum concentration of CBDA of 72.7 ng/mL was observed at 14 h after administration. The geometric mean half-life of CBDA was 14.1 h. No changes in serum biochemistry analysis were observed following IH dosing compared to baseline values. These results show acidic cannabinoids, especially CBDA, are readily absorbed from the rumen and available for distribution throughout the body.

## Introduction

Hemp has been grown and cultivated for centuries throughout the world. In the 1930’s, concerns about tetrahydrocannabinol intoxication in people resulted in regulation of hemp production by the Bureau of Narcotics and Dangerous Drugs and eventually the US Drug Enforcement Agency (DEA). However, the 2014 Farm Bill allowed for the research based cultivation and production of industrial hemp [*Cannabis sativa* containing < 0.3%Tetrahydrocannabinol (THC)] for fiber and phytocannabinoid production. The 2018 Farm Bill took the additional step of removing industrial hemp (IH) from the DEA list of Schedule I drugs renewing interest in hemp cultivation as a novel agricultural commodity. Hemp plants, and their by-products, are considered to have nutritional and potentially therapeutic value when used as a feed source in livestock. However, the absence of published data describing the disposition of bioactive phytocannabinoid compounds including cannabidiol (CBD), cannabigerol (CBG), tetrahydrocannabinolic acid (THCA-A), and cannabidiolic acid (CBDA) in edible tissues after hemp consumption is a significant impediment to future animal health and production research in this field.

Pharmacokinetic data for cannabinoids has been published in humans, mice, and swine^1–4^. These studies were aimed at providing toxicological data for illicit cannabis use or data from animal models for disease syndromes. There are no published studies investigating the pharmacokinetics of cannabinoids in ruminant species, such as cattle. Cattle can utilize industrial hemp byproducts as they can digest cellulose plant materials in their rumens. The objective of this study was to describe the pharmacokinetics of the cannabinoid, CBDA, and to describe the presences of other cannabinoids found in plasma after administration of industrial hemp with known cannabinoid content to cattle.

## Results

No adverse effects (reactions and behavioral changes) were observed following oral dosing of IH. The final mean (± SD) CBDA dose administered to the cattle was 5.4 ± 0.4 mg/kg. The total mg of each cannabinoid dosed are listed in Table 1. Only 5 of the 11 detected cannabinoids in the hemp plant material administered to the cattle were detected in the plasma following oral administration. Cannabidiol (CBD) was detected in four samples from two calves. The psychoactive cannabinoid 9-THC was not detected in any plasma samples. The cannabinoids ( ±)-cis-11-Nor-9-carboxy-Δ9-tetrahydrocannabinol glucuronide (THC-glu), ( ±)-11-Hydroxy-Δ9-tetrahydrocannabinol (THC-OH), , cannabidivarin (CBDV), Δ9-Tetrahydrocannabinolic acid A (THCA-A), cannabigerolic acid (CBGA), cannabigerol (CBG), Δ9-tetrahydrocannabinol (9-THC), Δ8-tetrahydrocannabinol (8-THC), cannabichromene (CBC), Δ9-tetrahydrocannabivarin (THCV), cannabinol (CBN), and (-)-11-nor-9-Carboxy-Δ9-tetrahydrocannabinol (THC-acid).were not detected in any sample at any time points.

**Table 1:** Individual animal weights (kg), total industrial hemp administered (g), and doses (mg) of individual cannabinoids administered to each animal.

	Animal ID								Mean	SD
	1	2	3	4	5	6	7	8		
Body weight, kg	205.9	200.0	250.9	253.6	205.0	219.5	191.8	191.4	214.8	23.2
Total hemp administered, g	30.7	34.8	39.0	37.8	33.2	34.2	34.9	33.3	34.7	2.5
*Cannabinoid doses, mg*										
Cannabidiolic acid (CBDA)	1010.0	1144.9	1283.1	1243.6	1092.3	1125.2	1148.2	1095.6	1142.9	81.1
Cannabidivarinic acid (CBDVA)	3.1	3.5	3.9	3.8	3.3	3.4	3.5	3.3	3.5	0.2
Tetrahydrocannabinolic acid-A (THCA-A)	103.7	117.6	131.8	127.7	112.2	115.6	117.9	112.5	117.4	8.3
Cannabichromenic acid (CBCA)	89.5	101.5	113.7	110.2	96.8	99.7	101.8	97.1	101.3	7.2
Cannabidiol (CBD)	107.7	122.1	136.9	132.6	116.5	120.0	122.5	116.8	121.9	8.6
9-Tetrahydrocannabinol (9-THC)	20.4	23.1	25.9	25.1	22.0	22.7	23.2	22.1	23.1	1.6
Cannabigerolic acid (CBGA)	59.5	67.4	75.6	73.3	64.3	66.3	67.6	64.5	67.3	4.8
Cannabigerol (CBG)	7.1	8.0	9.0	8.7	7.6	7.6	8.0	7.7	8.0	0.6
Cannabichromene (CBC)	15.7	17.9	20.0	19.4	17.0	17.5	17.9	17.1	17.8	1.3
Cannabinol (CBN)	0.8	0.9	1.1	1.0	0.9	0.9	0.9	0.9	0.9	0.1
Cannabidivarin (CBDV)	0.2	0.3	0.3	0.3	0.3	0.3	0.3	0.3	0.3	0.0

**Figure 1:**
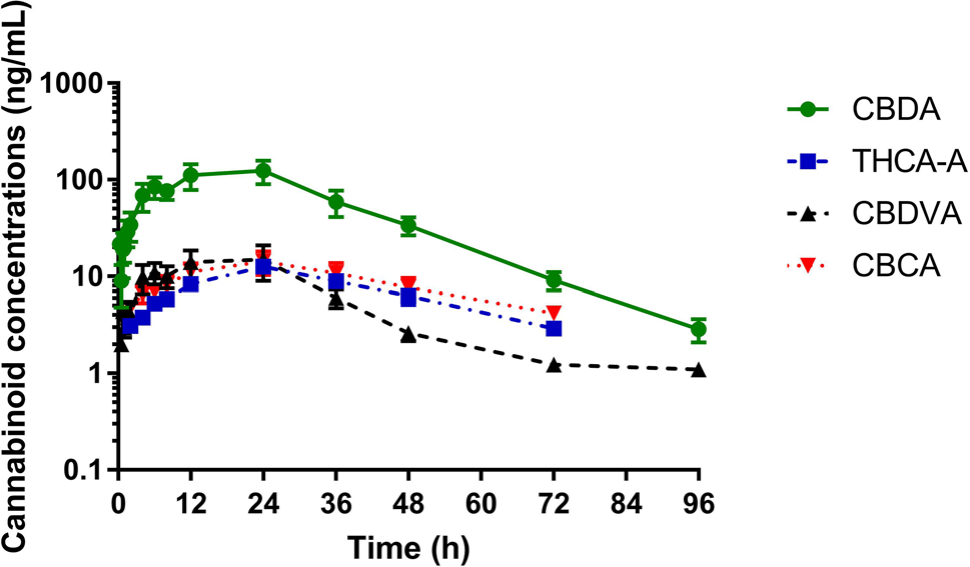
Mean plasma concentrations (ng/mL) of cannadibiolic acid (CBDA) at 5.4 mg/kg, tetrahydrocannabinolic acid-A (THCA-A) at 0.6 mg/kg, cannabidivarinic acid (CBDVA) at 0.02 mg/kg, and cannabichromenic acid (CBCA) at 0.5 mg/kg over time in cattle exposed to industrial hemp (n = 8).

Plasma CBDA, THCA-A, CBCA, and CBDVA concentrations over time are shown in Fig. 1. The pharmacokinetic parameters of CBDA are summarized in Table 2. Following oral administration of IH, CBDA reached a geometric mean maximum concentration of 72.7 ng/mL, which was observed at 11.8 h after dosing. A geometric mean apparent half-life (T½) of 14.1 h was recorded. The geometric mean AUC_0-∞_ was 2718.6 h × ng/mL. A geometric mean residence time of 30.6 h was determined. The arithmetic mean observed maximum concentrations of THCA-A, CBCA, and CBDVA were 12.1 ng/mL, 12.3 ng/mL, and 13.1 ng/mL respectively. The times of the mean maximum concentrations were observed at 25.2 h, 23.2 h, and 13.6 h for THCA-A, CBCA, and CBDVA.

**Table 2:** Pharmacokinetic parameters of cannabidiolic acid (CBDA) following oral administration of industrial hemp to achieve a 5.4 mg/kg dose (n = 8).

Parameter	Unit	Geometric mean	Median	Range
λ_z_	1/h	0.05	0.05	0.04–0.07
T1/2	h	14.1	13.89	9.62–19.59
Tmax	h	11.82	10	6.0–24.0
Cmax	ng/mL	72.7	74.7	36.5–177.0
AUC_0-∞_	h × ng/mL	2718.6	2995.8	1217.7–5714.6
AUC_0-last_	h × ng/mL	2593.9	2772.2	1099.7–5698.6
AUC extrapolated	%	3.0	3.1	0.3–11.2
AUMC_0-∞_	h^2^ × ng/mL	83,289.8	91,850	44,793.2–156,711.1
MRT_0-∞_	h	30.6	29.5	25.8–37.6
MRT_0-last_	h	27.6	27.0	23.4–32.1
Cl/F	mL/h/kg	1958.3	1955.0	927.5–4023.8
Vz/F	L/kg	39.7	42.3	12.9–107.2

Mean serum biochemical analysis are summarized in Table 3. Glucose and alkaline phosphatase (ALP) were elevated pre- and post-dosing. Additionally, blood urea nitrogen (BUN) were lower than reference ranges pre- and post-dosing. Other changes in the biochemical profiles of calves following IH administration were observed, but these changes remained within the normal limits of the tests. There were no significant changes in serum biochemical analytes observed.

**Table 3:** Results of serum blood chemistry profiles pre- and post-industrial hemp administration.

Parameter	Reference range	Unit	Pre-dose	Post-dose
			Mean 95% CI	Mean 95% CI
Glucose	29–73	mg/dL	102.9 98.9–106.8	98.2 94.3–102.2
BUN	9–24	mg/dL	2.8 2.2–3.3	2.6 2.1–3.2
Creatinine	0.5–1.6	mg/dL	0.8 0.7–0.8	0.7 0.6–0.7
Total protein	6.0–9.0	g/dL	6.5 6.3–6.6	6.4 6.2–6.5
Albumin	3.1–4.3	g/dL	3.6 3.5–3.8	3.6 3.4–3.7
Globulin	No ref interval	g/dL	2.8 2.6–3.0	2.8 2.6–3.0
Calcium (total)	8.1–10.3	mg/dL	10.8 10.5–11.1	10.6 10.3–10.9
Phosphorus	4.9–9.0	mg/dL	6.9 6.3–7.5	7.9 7.3–8.5
Sodium	138–155	mmol/L	141.8 140.2–143.3	141.0 139.4–142.6
Potassium	4.2–6.3	mmol/L	4.0 3.9–4.2	4.3 4.1–4.4
Chloride	92–117	mmol/L	95.3 93.9–96.6	97.6 96.2–98.9
Bicarbonate	21–36	mmol/L	29.4 27.5–31.2	31.0 29.2–32.8
Aspartate transaminase	53–156	U/L	77.3 70.0–84.5	81.3 74.0–88.5
Alkaline phosphatase	20–76	U/L	274.4 205.3–343.4	284.9 215.8–353.9
Gamma glutamyltransferase	10–39	U/L	11.0 9.2–12.8	10.0 8.2–11.8
Sorbitol dehydrogenase	6.1–18.4	U/L	20.5 18.4–22.6	17.4 15.3–19.5
Creatinine kinase	171–357	U/L	133.3 104.8–161.7	133.0 102.6–163.4

## Discussion

This is the first report describing the pharmacokinetics of cannabinoids in cattle after administration of industrial hemp. Specifically, this is the first report to focus on CBDA pharmacokinetics in cattle. Cannabidiolic acid is a precursor of cannabidiol (CBD), and present in high concentrations in industrial hemp. Cannabidiolic acid is converted to CBD via a decarboxylation reaction^5^.

In cattle, CBDA is absorbed from the rumen. The target dose of 5.0 mg/kg was chosen as it is the starting dose of cannabidiol for patients with seizures due to Lennox-Gastaut or Dravet syndromes^6^. The actual dose administered was 5.4 mg/kg or a mean of 1142.9 g per animal. This difference is due to the hemp boluses being prefilled and weighed prior to study commencement.

The pharmacokinetics of cannabinoids has been studied in mice as part of research to examine therapeutic control of seizures. Following intraperitoneal injection, cannabidiolic acid is rapidly absorbed reaching peak concentrations within 15 min; and has a relatively short half-life of 45 min. This is in contrast to cannabidiolic acid following oral dosing in cattle. In cattle, cannabidiolic acid is slowly absorbed from the rumen, and has a longer half-life. These pharmacokinetic data are needed to further design studies to investigate multiple daily doses, tissue residue kinetics, and eventual clinical studies to evaluate CBDA therapeutic and production effects in cattle.

The absorption of cannabinoids appears to be influenced by the chemistry of the cannabinoid. Acidic cannabinoids (CBDA, THCA-A, CBDVA, CBCA) were predominantly detected in the plasma after dosing. Cannabidiol (CBD) despite having a similar dose on a total mg basis as THCA-A, was only detected in four total samples. Interestingly, CBDVA was the lowest dosed cannabinoid on a total milligram basis (mean 3.5 mg per animal) yet it was found in all calves with maximum concentrations similar to THCA-A and CBCA. This suggests that CBDVA may have a higher oral bioavailability than the other compounds examined. However, a prospective study involving IV administration, is required to determine bioavailability. A similar pattern of the acidic forms of cannabinoids (CBDA and THCA-A) was observed in humans administered oral cannabis. Study participants were administered cannabis oil containing 2.3 mg/mL THCA-A, 2.2 mg/mL 9-THC, 4.4 mg CBDA, and 2.4 mg/mL CBD. The maximum concentrations (Cmax) of the four cannabinoids were 48.9 ng/mL, 1.4 ng/mL, 74.6 ng/mL and 4.4 ng/mL for THCA-A, 9-THC, CBDA, and CBD respectively^7^. These values are similar to others published for human cannabis users^8^.

Moreover, the impact of the rumen on the fate of oral cannabinoids requires further investigation. Rumen microbes could potentially degrade or metabolize cannabinoids causing alterations in the cannabinoids available for absorption. Merrick et al., reported the in vitro conversion of cannabidiol (CBD) to 9-tetrahydrocannabinol (9-THC) in simulated gastric fluid^9^. Although these findings were not supported in vivo^10^; there is still potential for rumen microbes to play a significant role in the conversion of fatty-acids through biohydrogenation.

Blood serum chemistry were taken prior to IH administration and 96 h after IH administration. There were no changes observed. The liver enzyme alkaline phosphatase was elevated in all animals prior to IH administration. Sorbitol dehydrogenase, a liver associated enzyme, was elevated prior to IH dosing, but returned to normal levels 96 h after dosing. However the change in sorbitol dehydrogenase was not statistically significant. Mean blood urea nitrogen (BUN) levels were below the reference range. This is likely due to the lower protein diet the calves were consuming. Further investigation into the impact of abnormal serum biochemical analytes is warranted, however the serum total proteins and albumin were within normal limits. The cattle in used in the study were on-site for five months prior to study commencement and were considered healthy on examination by a veterinarian.

The results of the finding of this study have implications for IH as an agriculture commodity. In the short-term, these findings can be used to develop strategies for cattle accidently exposed to IH and hemp by-products, as the U.S. Food and Drug Administration (FDA) has explicitly stated cannabinoids are considered adulterants in food production species. However, cattle and other ruminants are ideally suited to utilize IH and the byproducts of cannabinoid production from IH as a novel source of nutrition. Understanding of plasma half-lives for cannabinoids will allow veterinarians to work with cattle producers to establish withdrawal intervals to ensure exposed cattle can enter the food supply. Additionally, understanding of cannabinoid pharmacology is needed if IH and hemp byproducts are to be considered by the US FDA and the Association of American Feed Control Officials (AAFCO) for inclusion into animal diets.

The bulk of this report is centered on the pharmacokinetics of CBDA, but the absorption of THCA-A, CBCA, and CBDVA was observed. This work is an important first step towards follow-up studies that are needed to characterize cannabinoid absorption, distribution, metabolism, and excretion in cattle. Specifically, additional work is required to determine edible tissue residue profiles for cannabinoids in cattle. Evaluation of the role rumen microflora may have on the metabolism and degradation of cannabinoids administered to cattle either by direct exposure of specific cannabinoids or via hemp products is needed.

## Materials and methods

### Subjects and housing

Eight Holstein castrated males 10 months of age and weighing (± SD) 214 ± 23 kg were enrolled onto the study. Calves were acclimated to the research facility and halter trained for 1 week prior to study commencement. These calves were group housed in outdoor dirt pens with access to shelter. Pen size per calf exceeded the recommendations set forth in the Guide for the Care and Use of Agricultural Animals in Research and Teaching^11^. Calves were fed a custom grain mix at 8:00 and 16:00 h. Calves had ad libitum access to grass hay and water via self-filling trough for the entire duration of the study.

This study was performed in accordance with all relevant legislative and regulatory requirements in the United States, the State of Kansas, and AAALAC. All experimental procedures were approved by the Institutional Animal Care and Use Committee at Kansas State University (IACUC# 4326). Industrial hemp was grown and handled under license of the Kansas Department of Agriculture Industrial Hemp Research Program (licenses numbers: KDA-0621466839 and KDA-0302873296).

### Industrial hemp dosing and cattle sampling

Cannabinoid content of the IH was determined using ULPC-MS methods prior to the start of the study to determine cannabinoid dosage. As CBDA was the highest cannabinoid found in the IH, and considered the target cannabinoid for dosing. The target dose of CBDA was 5.0 mg/kg. For dosing, IH flower material was placed into gelatin capsules and the weight of each filled capsule was recorded. At the time of dosing cattle were administered four IH filled capsules. The weight of each capsule was recorded and a total weight of the IH administered was determined. Calves were monitored by study personnel for rejection (spitting out) the hemp boluses for 15 min following dose administration to confirm delivery.

Calves had a sterile jugular catheter placed 12 h prior to IH dosing to facilitate serial blood sampling for the first 12 h of the study. Blood samples for plasma cannabinoid analysis were obtained via the jugular catheter or jugular venipuncture prior to IH administration, and at 0.25, 0.5, 0.75, 1, 1.5, 2, 4, 6, 8, 12, 24, 36, 48, 72, and 96 h. Blood was placed into prelabeled vacutainer tubes containing lithium heparin and the contents mixed. After collection, whole blood was placed on ice and transported to the laboratory within 1 h of collection. Blood tubes were centrifuged at 1500 g for 10 min. Each plasma sample was harvested into individual cryovials and placed in − 80 °C freezer until analyzed.

Additionally, prior to IH dosing and at 96 h, blood was obtained and placed into vacutainers with no additive for serum biochemical analysis. Serum samples were submitted to the Kansas State Veterinary Diagnostic Laboratory for biochemical analysis by photometric methods (Cobas 501, Roche Diagnostics, Indianapolis, IN, USA).

### Cannabinoid analysis

Plasma cannabinoid concentrations were determined using UPLC-MS methods adapted from Zhang et al.^12^. All solvents used such as methanol, acetonitrile, isopropanol, formic acid were LC–MS grade. Cannabinoids standards were purchased in individual solutions in methanol (Cerilliant Corporation, Round Rock, TX), including: ( ±)-cis-11-Nor-9-carboxy- Δ 9-tetrahydrocannabinol glucuronide (THC-glu), ( ±)-11-Hydroxy-Δ9-tetrahydrocannabinol (THC-OH), cannabidivarinic acid (CBDVA), cannabidivarin (CBDV), cannabidiol (CBD), cannabidiolic acid (CBDA), Δ9-Tetrahydrocannabinolic acid A (THCA-A), cannabigerolic acid (CBGA), cannabigerol (CBG), Δ9-tetrahydrocannabinol (9-THC), Δ8-tetrahydrocannabinol (8-THC), cannabichromene (CBC), Δ9-tetrahydrocannabivarin (THCV), cannabichromenic Acid (CBCA), cannabinol (CBN), (-)-11-nor-9-Carboxy-Δ 9-tetrahydrocannabinol (THC-acid). Cannabinoid analogs used as internal standards were also purchased in solution in methanol at 100 µg/mL (Cerilliant Corporation, Round Rock, TX): ( ±)-cis-11-Nor-9-carboxy-Δ9-tetrahydrocannabinol glucuronide (THC-glu-d_3_), Cannabidiol-d_3_ (CBD-d_3_), Δ9-Tetrahydrocannabinol-d_3_ (9-THC-d3), ( ±)-11-nor-9-Carboxy-D9-tetrahydrocannabinol-d_9_ (THC-acid-d_9_), ( ±)-11-Hydroxy-Δ9-tetrahydrocannabinol-d_3_ (THC-OH-d_3_), Cannabichromene-d_9_ (CBC-d_9_). All cannabinoids standards were kept in the freezer at − 20 °C.

On the day of analysis, plasma samples were allowed to thaw. Once thawed, 0.1 mL of plasma (samples, quality controls or negative control plasma) were mixed with 0.1 mL of internal standard mixture at 200 ng/mL (not added to the negative control sample) and 0.1 mL of acetonitrile with 0.1% formic acid to each samples to precipitate the proteins. The mixture was vortexed for 5 s and centrifuge for 5 min at 7000 g. The supernatant was then diluted with 0.4 mL of ultra-pure 18 Ω water before clean-up. The sample was loaded on a solid phase extraction plate using positive pressure nitrogen. Each well was washed twice with 0.25 mL of a mixture of methanol–water (25:75). The compounds were eluted with two-25 µL aliquots of acetonitrile-methanol (90:10) and diluted with 50 µL of water before analysis.

Cannabinoid analysis was performed using an Acquity H UPLC and a TQ-S triple quadrupole mass spectrometer (Waters Corp., Milford, MA). The chromatographic separation was performed with a UPLC column (Eclipse Plus C18, Agilent Technologies, Santa Clara, CA) 100 × 2.1 mm, 1.8 µ, heated at 55 °C. The flow rate was set at 0.5 mL/min, the mobile phase consisted of a gradient of acetonitrile (B) and water containing 0.1% formic acid (A) as follow: 0 min: 60%B, 6.50 min: 86% B, 7.50 min-9 min: 100%B, 9.01 m in12 min: 60%B. The total run time was 12 min. The injection volume was 5 µL. The data acquisition was performed by electrospray ionization in positive and negative mode using multiple reaction monitoring. The capillary voltage was 3.0 kV, the source temperature 150 °C, the desolvation temperature 500 °C, the desolvation nitrogen flow at 1000 L/h, and the cone nitrogen flow at 150 L/h. Linear regression with a weighing factor of 1/X was used and accepted if the coefficient of correlation R^2^ was > 0.99. Calibration curves were linear from 0.1 to 100 ng/mL for all cannabinoids. The lower limit of detection, lower limit of quantification, intra-day precisions, inter-day precisions, and inter-day accuracies for each cannabinoid analyte are summarized in Table 4.

**Table 4:** Lower level of detection (LOD), lower limit of quantification (LOQ), intra-day precisions, inter-day precisions, and inter-day accuracies for each cannabinoid analyte analyzed following oral administration of industrial hemp.

Cannabinoid analytes	LOD	LOQ	Intra-day precision (n = 3)			Inter-day precision (n = 6)			Inter-day accuracy (n = 6)		
			1.75	47.5	95.0	1.75	47.5	95.0	1.75	47.5	95.0
Tetrahydocannabinol-glucuronide (THC-glc)	0.2	0.5	1.5	3.0	4.5	10.5	8.6	7.2	106	105	106
Cannabidivarinic acid (CBDVA)	0.2	1.0	12.3	7.8	14.7	32.5	33.8	33.6	121	115	119
Hydroxy-tetrahydrocannabinol (THC-OH)	0.2	0.25	2.2	6.1	3.7	7.8	5.7	5.8	110	103	103
Tetrahydrocannabinolic-acid (THC-acid)	0.2	0.5	5.7	2.4	4.0	4.2	3.5	5.2	112	103	103
Cannabidivarin (CBDV)	0.2	0.25	4.3	2.6	4.0	6.8	8.5	6.6	105	99	99
Cannabichromenic acid (CBCA)	1.0	2.5	16.7	9.2	6.8	24.7	29.2	24.3	105	99	99
Tetrahydrocannabivarin (THCV)	0.2	0.25	4.3	3.4	3.0	4.5	5.8	6.0	105	110	99
Cannabigerol (CBG)	0.2	0.25	4.8	3.0	2.5	8.3	10.5	12.0	107	100	95
Cannabidiol (CBD)	0.2	0.5	8.4	3.5	4.2	6.9	4.6	6.0	113	103	101
Cannabinol (CBN)	0.2	0.5	1.5	3.4	3.6	5.8	5.2	5.6	105	97	95
9-Tetrahydrocannabinol (9-THC)	0.2	0.5	4.0	3.1	4.1	4.0	3.3	4.0	110	104	103
8-Tetrahydrocannabinol (8-THC)	0.2	0.5	6.3	4.0	3.6	5.3	5.5	5.3	104	100	101
Cannabichromene (CBC)	0.2	0.5	3.8	2.4	2.1	3.3	3.6	3.9	112	103	103
Tetrahydrocannabinolic acid-A (THCA-A)	1.0	2.5	8.5	3.7	8.0	18.2	5.3	8.7	100	89	95
Cannabichromenic acid (CBCA)	1.0	2.5	3.6	4.7	9.3	20.3	7.3	10.9	113	100	108

*LOD* lower limit of detection, *LOQ* lower limit of quantification.

### Pharmacokinetic analysis

Individual animal pharmacokinetic parameters were calculated for CBDA concentration vs. time data using computer software (Phoenix 8.2, Certara, Inc., Princeton, NJ, USA). Non-compartmental approach based on statistical moment theory was used for the analysis. The area under the plasma concentration versus time curve (AUC) and area under the first moment of the concentration vs time curve (AUMC) were determined using the linear trapezoidal linear interpolation method. The AUC_0_-_last_ and AUMC_0-last_ of CBDA, the time range from the time of drug administration to the last quantifiable drug concentration were extrapolated to infinity to determine AUC_0_-_∞_, AUMC_0_-_∞_. The mean residence times (MRT_0_-_∞_) of CBDA following administration was calculated from AUC_0_-_∞_ and AUMC_0_-_∞_ (i.e., MRT_0_-_∞_ = AUMC_0_-_∞_/AUC_0_-_∞_). The elimination rate constants (λ_z_) associated with the terminal elimination phase was estimated by using linear regression of the terminal phase of the log plasma concentration vs. time curve. The elimination half-lives (λ_z_-HL) were calculated using the equation = $\frac{0.693}{{\lambda }_{z}}$. The maximum plasma concentration (C_max_) and time to obtain maximum concentration (T_max_) were reported as observed values. The apparent volume of distribution per fraction of the dose absorbed based on the terminal phase (V_z_/F) and apparent total body clearance per fraction of dose absorbed (CL/F) were calculated. Pharmacokinetic parameters are presented as geometric means, medians, and ranges based on the consideration of log-normal distributions.

## Data Availability

All data generated or analyzed for this study are included in this article.
